# Duodenal Cytochrome *b* (DCYTB) in Iron Metabolism: An Update on Function and Regulation

**DOI:** 10.3390/nu7042274

**Published:** 2015-03-31

**Authors:** Darius J. R. Lane, Dong-Hun Bae, Angelica M. Merlot, Sumit Sahni, Des R. Richardson

**Affiliations:** Molecular Pharmacology and Pathology Program, Department of Pathology and Bosch Institute, University of Sydney, Sydney, NSW 2006, Australia; E-Mails: dbae5413@uni.sydney.edu.au (D.-H.B.); angelica.merlot@sydney.edu.au (A.M.M.); sumit.sahni@sydney.edu.au (S.S.)

**Keywords:** DCYTB, *CYBRD1*, vitamin C, cytochrome *b*_561_, iron homeostasis, anemia, mouse model, HIF2α, IRP1

## Abstract

Iron and ascorbate are vital cellular constituents in mammalian systems. The bulk-requirement for iron is during erythropoiesis leading to the generation of hemoglobin-containing erythrocytes. Additionally, both iron and ascorbate are required as co-factors in numerous metabolic reactions. Iron homeostasis is controlled at the level of uptake, rather than excretion. Accumulating evidence strongly suggests that in addition to the known ability of dietary ascorbate to enhance non-heme iron absorption in the gut, ascorbate regulates iron homeostasis. The involvement of ascorbate in dietary iron absorption extends beyond the direct chemical reduction of non-heme iron by dietary ascorbate. Among other activities, intra-enterocyte ascorbate appears to be involved in the provision of electrons to a family of trans-membrane redox enzymes, namely those of the cytochrome *b*_561_ class. These hemoproteins oxidize a pool of ascorbate on one side of the membrane in order to reduce an electron acceptor (e.g., non-heme iron) on the opposite side of the membrane. One member of this family, duodenal cytochrome *b* (DCYTB), may play an important role in ascorbate-dependent reduction of non-heme iron in the gut prior to uptake by ferrous-iron transporters. This review discusses the emerging relationship between cellular iron homeostasis, the emergent “IRP1-HIF2α axis”, DCYTB and ascorbate in relation to iron metabolism.

## 1. Introduction

Iron is critical for cell survival, as demonstrated by cell death following cellular iron-depletion [1,2]. Adult humans contain 3–5 grams of iron, up to 80% of which is found in erythrocyte hemoglobin, while a further 20% is stored transiently within macrophages and hepatocytes [3,4]. Cellular iron storage typically occurs within the intracellular iron storage protein, ferritin [5]. The remainder of the iron is present in other heme-containing proteins (e.g., cytochromes), iron-sulfur cluster (ISC)-containing proteins (e.g., succinate dehydrogenase) [6,7,8] and non-heme/non-ISC iron-containing proteins (e.g., iron- and 2-oxoglutarate-dependent dioxygenases) [9,10]. Approximately 20 mg of iron is required daily for the *de novo* production of hemoglobin, 90%–95% of which is derived from iron that is recycled from senescent and effete erythrocytes by reticuloendothelial macrophages in the spleen. Approximately 1–2 mg of iron is absorbed in the proximal duodenum to replace iron losses from bleeding, desquamation of epithelial cells, sweating and urinary excretion [4].

Iron in biological systems is typically capable of one-electron redox cycling between its ferric and ferrous forms. While this activity endows iron with much of its biological utility, iron that is not bound in a redox-inert form can catalyze the production of noxious reactive oxygen species (ROS) through Fenton and Haber-Weiss-type reactions [3,11]. As a consequence, iron homeostasis is tightly controlled through regulating its import, storage and cellular efflux [3,12,13,14].

Ascorbate is a co-factor in numerous metabolic reactions [15]. Humans cannot synthesize ascorbate due to a ubiquitous inactivation of the gene encoding the enzyme, l-gulono-γ-lactone oxidase (GULO), which is essential for ascorbate synthesis [16]. In addition to ascorbate’s capacity to stimulate dietary iron absorption [17], this vitamin also affects iron metabolism by stimulating ferritin synthesis [18] and by inhibiting lysosomal ferritin degradation [19,20] and cellular iron efflux [21]. Further, we have recently shown that the release of ascorbate from ascorbate-replete cells is responsible for ascorbate-stimulated iron uptake from low-*M_r_* iron-citrate complexes [15,22,23], which are prominent in plasma of patients with iron-overload disorders [24].

Additionally, ascorbate appears to be involved in the provision of electrons to a family of *trans*-membrane redox enzymes, namely those of the cytochrome *b*_561_ class. These hemoproteins oxidize a pool of ascorbate on one side of the membrane in order to reduce an electron acceptor (e.g., non-heme iron) on the opposite side of the membrane. One member of this family, duodenal cytochrome *b*_561_ (DCYTB/CYBRD1/CYB561A2), may play an important role in ascorbate-dependent reduction of non-heme iron in the gut prior to uptake by ferrous-iron transporters.

The remainder of this review provides an overview of the intestinal absorption of dietary non-heme iron in relation to systemic and cellular iron homeostasis. This discussion will focus on the proposed physiological functions for the intestinal ferrireductase, DCYTB, and the current evidence for a critical role for this reductase in dietary iron absorption. Finally, we examine the emerging iron regulatory protein 1 (IRP1)-hypoxia-inducible factor 2α (HIF2α) axis and its ability to regulate expression of DCYTB, and other key iron absorption and metabolism proteins.

### 1.1. Systemic Iron Homeostasis—An Overview

Systemic iron homeostasis is largely controlled at the level of iron efflux from three key cell-types into the circulation: (i) dietary iron absorption by duodenal enterocytes (*i.e.*, via reduced iron efflux into the portal circulation); (ii) iron recycling by reticuloendothelial macrophages (*i.e.*, by decreased iron efflux into the plasma following the phagocytic turnover of effete erythrocytes); and (iii) iron release from hepatocytes.

Iron that is released into the circulation by duodenal enterocytes, reticuloendothelial macrophages and hepatocytes is exported by the iron export protein, ferroportin (FPN1) [25]. This iron exporter appears to transport iron in the ferrous form, which helps explain the requirement for ferroxidase activity accompanying iron efflux and iron-loading of transferrin [26]. The amount of FPN1 in major iron-releasing cell-types is modulated in response to iron by multiple mechanisms, including transcriptional and post-transcriptional regulation, and at the level of ligand-induced protein degradation within the autolysosome [27]. The latter is regulated by a key hormone of iron metabolism, hepcidin, which is predominantly produced and secreted by hepatocytes [28]. The binding of hepcidin to FPN1 at the cell surface causes internalization and lysosomal degradation of the hepcidin-FPN1 complex [29]. Interestingly, in the plasma, hepcidin circulates bound specifically to α_2_-macroglobulin and non-specifically to albumin [30]. The binding of relatively small hepcidin peptide to α_2_-macroglobulin appears to decrease its urinary excretion [31].

The expression of hepcidin is enhanced by iron loading as well as certain inflammatory cytokines, and is suppressed by: iron deficiency and anemia, enhanced erythropoiesis and hypoxia [32]. For recent and comprehensive reviews on the regulation of hepcidin expression and the mechanism of its action, see Ganz and Nemeth [32], and Kautz and Nemeth [33].

### 1.2. Dietary Iron Absorption—An Overview

There is no regulated pathway for iron excretion from the body, and consequently, whole-body iron levels are predominantly controlled at the level of dietary iron absorption from the intestine [34]. Iron absorption occurs mainly in the duodenum (and upper jejunum) and is increased during iron deficiency and decreased during iron repletion and overload [4,35]. At the cellular level, iron is absorbed across the apical membrane of differentiated epithelial cells (enterocytes) of the mid and upper zones of the villus. These cells can absorb dietary iron, which comes in two major forms: non-heme iron, which is mainly found in cereals and vegetables; and heme iron, which is mainly sourced from hemoproteins in meat (for comprehensive reviews of intestinal iron absorption, see Sharp [36] and Gulec *et al.* [34]). The absorption of heme iron is more efficient than non-heme iron, but the mechanisms remain poorly understood, and will not be reviewed further here. 

The uptake of dietary non-heme iron occurs in two phases. In the first phase, iron is taken up across the apical membrane of duodenal enterocytes, which requires that the iron is both soluble and in its reduced form [37]. Dietary non-heme iron, which is typically in the form of low-*M*_r_ chelates of ferric iron, is typically liberated as low-*M*_r_ iron within the acidic environment of the stomach. The acidic chyme is then expelled from the stomach into the duodenum, where the majority of iron absorption is thought to occur. The current and widely-held view is that the reduction of non-heme iron, which is rapidly oxidized into its ferric form in the presence of dissolved oxygen, is mediated by an apical membrane ferrireductase, such as DCYTB [38]: a di-heme transmembrane oxidoreductase that appears to utilize intracellular ascorbate as an electron donor to reduce extracellular ferric iron and other physiological substrates (discussed further below) [15,39,40]. Importantly, there is also evidence that the reduction of non-heme iron in the extracellular environment may involve non-enzymatic ferrireduction driven by endogenous and secreted reductants [37], such as ascorbate [22,23,41] and/or superoxide [42,43]. The contribution of non-enzymatic reduction of duodenal iron by dissolved ascorbate, whether secreted or supplied in the diet, may be of pathophysiological relevance to the mechanism of iron reduction and absorption in mammals (e.g., humans) that are incapable of synthesizing their own ascorbate.

Ferrous iron is then transported across the apical membrane of the enterocyte via a ferrous iron transporter, such as divalent metal transporter 1 (DMT1) [44], and possibly other divalent metal transporters, such as ZIP14 [45]. Iron that reaches the basolateral membrane by poorly understood mechanisms can then be transported into the circulation by FPN1 [25,46]. The *trans*-membrane ferroxidase, hephaestin, colocalizes with FPN1 in the basolateral membrane and, in combination with plasma ceruloplasmin, helps oxidize the exported ferrous iron back to ferric iron [47,48]. This iron is then complexed to the major plasma iron transport protein, transferrin (Tf), for transport through the circulation and uptake by distal tissues. Additionally, Tf-bound iron is virtually the sole source of iron for the erythropoietic compartment that is responsible for the daily production of approximately 200 billion erythrocytes [3].

## 2. DCYTB and Ascorbate: A Critical Link in Iron Metabolism?

As recently reviewed [49], in addition to its many roles in cellular physiology, ascorbate is emerging an important modulator of cellular iron metabolism. Indeed, accumulating evidence indicates that, in addition to the known ability of dietary ascorbate to enhance non-heme iron absorption in the gut (see above), ascorbate can actively regulate cellular iron uptake and downstream cellular iron metabolism. In fact, ascorbate is known to regulate iron metabolism by: (i) increasing Tf-dependent iron uptake, possibly via an endosomal reductive mechanism [50], and increasing non-Tf-bound iron uptake, the latter of which occurs by a *trans*-plasma membrane ascorbate/DHA cycle [22,23,41,50]; (ii) increasing and/or maintaining the levels of IRP1 in its non-iron responsive element (IRE)-binding cytosolic aconitase form, which leads to an increase in ferritin synthesis [18,51]; and (iii) inhibiting lysosomal ferritin degradation via autophagy [19].

Additionally, ascorbate is capable of modulating FPN1 [52] and/or cellular iron efflux activity [21,52]. Indeed, we have shown that ascorbate can decrease iron efflux from various cell-types [21,50], although, whether this iron-efflux is FPN1-dependent is unclear. Considering this, it is notable that near-physiological extracellular ascorbate levels (100 μM) caused an increase in FPN1 expression in intestinal epithelial-like human Caco-2 cells, which was associated with an increase in IRP2 and HIF2α [52]. Whether the increase in FPN1 was due to an ascorbate-dependent increase in ISC formation/retention within IRP1 [51], which could explain the increase in HIF2α and subsequent increase in FPN1 (see below), is unclear.

As mentioned above, the other major role of ascorbate in iron metabolism appears to be through its activity in supplying reducing equivalents to DCYTB, a cellular ferrireductase, and perhaps other members of the cytochrome *b*_561_ family. The potential physiological functions of DCYTB are reviewed below.

### 2.1. The Cytochrome b_561_ Family and DCYTB

The cytochrome *b*_561_ family exists in all eukaryotic kingdoms [53]. For an excellent recent review, see Asard *et al.* [39]. Cytochrome *b*_561_ (also known as chromaffin granule cytochrome *b*_561_; CYB561/CYB561A1) catalyzes *trans*-membranous electron transfer from cytosolic ascorbate to intra-vesicular ascorbyl radicals in neuroendocrine secretory granules [54].

CYB561 was initially recognized as a redox-active component of catecholamine storage granules in the early 1960s [55]. This activity was originally demonstrated to be the result of a unique heme-containing cytochrome located in the membranes of these vesicles [56,57,58]. In the 1980s, spectroscopic and EPR studies demonstrated that this cytochrome was responsible for equilibrating the ascorbate-ascorbyl radical pair inside the granule lumen with this redox pair in the cytosol [59,60,61]. Indeed, CYB561 is canonically involved in trans-membranous electron transfer from cytosolic ascorbate to the intravesicular ascorbyl radical in neuroendocrine secretory granules [54,62,63]. The ascorbate that results from this redox reaction can then be oxidized by copper-containing dopamine β-hydroxylase and peptidyl glycine α-amidating monooxygenase [64]. The CYB561-catalyzed reaction involves a histidine cycling mechanism of coupled proton/electron transfer between ascorbate and the ascorbyl radical [65]. A recent atomic level crystal structure of *Arabidopsis thaliana* cytochrome *b*_561_ indicates this protein may function as a dimer [66]. Moreover, two highly conserved amino acids, Lys-81 and His-106, play vital roles in substrate recognition and catalysis [66]. Indeed, the structural and biochemical analyses presented in this study probably provide for a more general electron transfer mechanism that is applicable to other cytochromes *b*_561_ [66].

**Figure 1:**
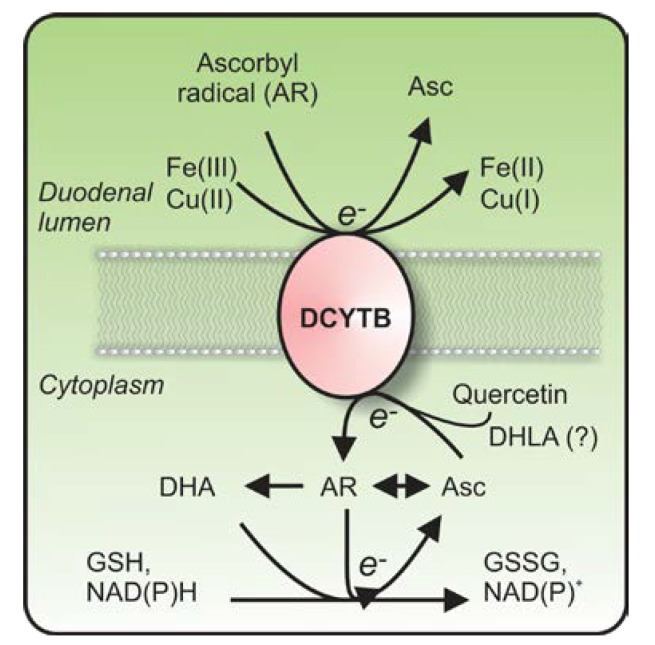
The role of duodenal cytochrome *b*_561_ (DCYTB) as a transplasma membrane oxidoreductase. DCYTB is transplasma membrane oxidoreductase that reduces either extracellular ferric iron, (Fe(III)), cupric copper (Cu(II)), or the ascorbyl radical (AR), at the expense of intracellular reducing equivalents derived proximally from intracellular ascorbate and/or quercetin, or possibly dihydrolipoic acid (DHLA). The source of reducing equivalents for the recycling of intracellular ascorbate from dehydroascorbate (DHA) or AR includes reduced glutathione (GSH), which becomes oxidized to GSSG, and/or NAD(P)H, which becomes oxidized to NAD(P)^+^, but also the mitochondrial respiratory chain (not shown). For a review on intracellular ascorbate recycling, see Linster [16] and Lane and Lawen [15].

In addition to DCYTB, additional members of cytochrome *b*_561_ family of enzymes in mammals include lysosomal cytochrome *b*_561_ (CYB561A3) [53,67], stromal cell-derived receptor 2 (SDR2/FRRS1) [68,69] and the putative tumor suppressor, 101F6 (CYB561D2/TSP10) [70,71]. Intriguingly, all proteins have been shown capable of stimulating cellular ferrireduction, and are expressed at the plasma membrane, as well as at intracellular sites [38,53,68,72,73]. As with CYB561, these proteins may also be involved in the *trans*-membrane reduction of the ascorbyl radical with cytosolic ascorbate as the electron donor [39] (Figure 1).

### 2.2. What Is the Identity of the Electron Donor for DCYTB?

While the bulk of evidence suggests that ascorbate is the major electron donor for many of the cytochromes *b*_561_ [39], a recent report suggests that dihydrolipoic acid can donate electrons to some cytochromes *b*_561_ under certain conditions [74]. However, there is no direct evidence that dihydrolipoic acid can donate electrons to DCYTB. Although the cellular levels of dihydrolipoic acid are relatively low compared to ascorbate, this activity may be partially responsible for an apparent ascorbate-independent ferrireductase activity of DCYTB [73]. Dihydrolipoic acid may also be involved in intracellular ascorbate recycling [75,76], so the contribution of this molecule to the activity of DCYTB, and other cytochromes *b*_561_, could potentially occur at multiple levels.

As DCYTB demonstrates partial conservation of the canonical ascorbate-binding motif originally identified in CYB561 [39,53,64], ascorbate is a likely proximal electron donor for DCYTB-dependent oxidoreduction. Accordingly, several studies in which DCYTB-expressing cells have been supplemented with ascorbate or DHA provide support for this conclusion [53,72,77,78,79]. However, one study has suggested that ascorbate is not essential, at least in airway epithelial cells [73].

The polyphenolic flavonoid, quercetin, has also been described to function as an electron donor for DCYTB [80]. This finding is significant given that quercetin is considered to be the most prevalent flavonoid in the Western diet [81]. This suggests that quercetin could be a quantitatively significant enterocytic reductant involved in the supply of electrons for DCYTB-dependent ferrireduction in the duodenum. Moreover, quercetin can be imported by facilitative glucose transporter (GLUT) 1 and 4 [82], and reversibly inhibits both glucose and DHA uptake by GLUTs 1–4 [83,84]. An additional consideration here is that quercetin-iron chelates appear to be able to shuttle iron in either direction across the plasma membrane via GLUT1 [85]. This suggests that quercetin is an important dietary factor that may be able to modulate dietary iron absorption by both DCYTB-dependent and -independent mechanisms. An interesting further possibility, which remains to be assessed, is that DCYTB reduces at least some iron in the form of quercetin-iron chelates in the duodenum.

### 2.3. DCYTB: Clarifying Its Role in Iron Metabolism

DCYTB is an iron-regulated ferrireductase that is highly expressed in the apical membrane of duodenal enterocytes [38,40]. This di-heme-protein [78,86] was originally suggested to be responsible for non-heme iron reduction during dietary iron absorption [38,77]. Indeed, it is highly expressed in the brush border membrane of the duodenal microvilli, where it appears to be regulated by iron, hypoxia, erythroid activity and by increased systemic iron requirements [38,40]. Emerging evidence also suggests that a promoter single nucleotide polymorphism in the DCYTB gene is a genetic modifier for the iron overload disorder, HFE hereditary hemochromatosis, possibly leading to a 30% decrease in promoter activity [87].

Importantly, DCYTB was identified by a subtractive cloning strategy, similar to that used to identify DMT1 [44] and FPN1 [46]. A duodenal ferrireductase had long been suspected to be required for dietary non-heme iron absorption. This notion was subsequently challenged by Gunshin and colleagues with the observation that *Cybrd1^−/−^* (*i.e.*, DCYTB-knockout) mice did not develop iron deficiency on a standard lab diet, or develop greater iron-deficiency on an iron-deficient diet, compared to wild-type mice [88]. The results of the Gunshin *et al.* [88] study are intriguing and are consistent with the likelihood that the activity of DCYTB may be supplemented by the action of other ferrireductases or ferrireductants. Indeed, a likely contributing factor to the apparent lack of phenotype in DCYTB-knockout is the presence of secreted ascorbate in gastric juice [40,89]. Indeed, ascorbate is a normal constituent of such secretions, even in ascorbate auxotrophs, such as humans [90]. As previously suggested, the levels of ascorbate secreted into the gut lumen may be markedly higher in mice and other species capable of synthesizing their own ascorbate [72,89]. Intriguingly, luminal ascorbate levels in the duodenum and duodenal mucosa have been shown to be regulated by iron, as they are increased in iron deficient and hypotransferrinaemic mice as well as by iron deficiency in humans [89,91].

An important caveat to study of Gunshin *et al.* [88] is that the results obtained do not directly pertain to the role of DCYTB in dietary iron absorption [92]. That is, although the inactivation of DCYTB does not alter hepatic iron levels of mice fed on a standard diet over the time-course examined, the standard chow employed contains high amounts of ferrous iron [92]. If DCYTB acts as a ferrireductase in the duodenum that is required to reduce ferric to ferrous iron prior to uptake by DMT1, the requirement for the activity of this protein under such conditions may be minimized. As previously suggested [92], additional important information would be obtained by examining the effect on hepatic iron stores of a diet containing exclusively ferric iron. Moreover, the role of DCYTB in iron absorption should be directly assessed through radioactive tracer studies in which normal or iron-deficient chow is supplemented with radioactive ferric iron [92].

As discussed further below (Section 3.3), DCYTB is strongly upregulated by hypoxia in a HIF2α-dependent manner. This suggests that the physiological significance of DCYTB in dietary iron absorption may be more apparent under conditions of hypoxia compared to normoxia. Indeed, a recent study from McKie’s group assessed the contribution of murine DCYTB to total duodenal ferric reductase activity, as well as to whole-animal iron metabolism, in the context of hypoxia [93]. They found that DCYTB was likely to be the only hypoxia-inducible ferrireductase in the duodenum, as there was no evidence of hypoxia-inducible ferrireductase activity in *Cybrd1*^−/−^ mice. Interestingly, while hematological indices and liver non-heme iron levels were unaffected in wildtype compared to *Cybrd1*^−/−^ mice under conditions of hypoxia, spleen non-heme iron levels in the *Cybrd1*^−/−^ animals were almost half that of the wildtype animals [93]. Additionally, under conditions of normoxia, *Cybrd1*^−/−^ mice exhibited evidence of impaired reticulocyte hemoglobinization [93]. These findings strongly suggest that DCYTB is the primary iron- and hypoxia-regulated duodenal ferrireductase, and that this protein is required for optimal iron absorption and systemic iron metabolism.

### 2.4. Does DCYTB Play a Role in the Reduction of Copper and Ascorbyl Radicals?

DCYTB has also been suggested to function as an oxidoreductase in a variety of tissue and cell-types [38,72,77,78]. These include human erythrocytes [94], airway epithelial cells [73], K562 cells [95,96,97], Hep-G2 cells [96], Caco-2 cells [79,96], and astrocytes [98,99]. This suggests a more general function for DCYTB than one of just enhancing dietary non-heme iron absorption. Several important studies have implicated DCYTB as a more general oxidoreductase, potentially involved in the cellular reduction of not only non-transferrin-bound iron and non-heme iron, but also copper and ascorbyl radicals. These studies are briefly reviewed below.

A role for an apparent DCYTB homologue in rabbit duodenal enterocytes [100] as a cell-surface cupric reductase has been proposed [72]. Indeed, MDCK cells heterologously and inducibly expressing DCYTB-EGFP at the plasma membrane demonstrated an enhanced ability to reduce both ferric and cupric ions [72]. These cells also endogenously express DMT1 and demonstrated an increased rate of ^59^Fe uptake from low-*M*_r_^59^Fe-NTA or ^59^Fe-citrate [72], the latter of which is a physiological low-*M*_r_ iron chelate [3]. As dietary copper must also be in the reduced form to be absorbed [101], these findings suggest that DCYTB may also act as an intestinal copper reductase that helps to augment dietary copper absorption by supplying cuprous ions for uptake by either copper transporter 1 [102] or DMT1 [103,104].

Copper and iron metabolism are intimately connected [101,105], particularly via the two multicopper ferroxidases, hephaestin and ceruloplasmin. Indeed, a recent study has demonstrated that copper deficiency leads to anemia, duodenal hypoxia, and the up-regulation of HIF2α and HIF2α-regulated iron absorption genes in mice (e.g., *Cybrd1*, *Dmt1* and *Fpn1*) [105]. Moreover, iron deficiency in mice leads to up-regulation of copper absorption genes (*Atp7a* and *Mt1*) [106]. Indeed, the enterocyte ATP-dependent copper exporter, Atp7a, is upregulated at the transcriptional level by HIF2α in intestinal epithelial cells [107]. A more recent study suggests that HIF2α-dependent upregulation of Atp7a during iron deficiency/hypoxia also requires the transcription factor, Sp1 [106]. Thus, the putative activity of DCYTB as a cupric reductase suggests a further connection between iron and copper metabolism, as well as a connection between ascorbate and copper metabolism. The role of HIF2α in regulating intestinal iron absorption will be discussed in more depth in Section 3.3.

Another important physiological electron acceptor for DCYTB appears to be the ascorbyl radical [94], which is the one-electron oxidized form of ascorbate. DCYTB also shows conservation of the predicted ascorbyl radical binding site, suggesting that it may be an additional native electron acceptor. Indeed, in similarity to CYB561, the putative DCYTB ascorbate-binding site maps to the cytosolic side of the membrane, while that of the ascorbyl radical binding site maps to the extracellular face [53,64]. This at least indicates that DCYTB-dependent catalysis of ascorbate:ascorbyl radical reduction is topologically plausible. Indirect experimental support for DCYTB-dependent ascorbyl radical reduction comes from an important study that identified DCYTB in human erythrocyte membranes [94]. Indeed, Su *et al.* [94] also determined that DCYTB occurs in erythrocyte membranes from guinea pigs, which, like humans, cannot produce their own ascorbate, but not erythrocyte membranes of rats and mice. Critically, both rats and mice are able to produce their own ascorbate, suggesting that the existence of DCYTB in the erythrocyte membrane is determined by the lack of ascorbate biosynthetic ability of that animal, and potentially, circulating ascorbate levels. Importantly, DCYTB-containing human erythrocytes were much more effective at preserving extracellular ascorbate than mouse erythrocytes. These findings suggest that the presence of DCYTB in erythrocytes from ascorbate auxotrophs (e.g., humans and guinea pigs) is an important adaptation allowing for enhanced conservation of plasma ascorbate.

As discussed previously [15], an important consideration here is whether the putative ability of DCYTB to facilitate ascorbyl radical reduction plays a part in DCYTB-dependent ferrireduction or copper reduction. That is, in the presence of ascorbate, DCYTB may act to facilitate ferrireduction or copper reduction by regenerating extracellular ascorbate, the latter of which has been shown to be effective in facilitating the direct and rapid reduction of iron prior to its cellular uptake [15,22,23,41,97]. In addition to ascorbate cycling across the plasma membrane [22,23,41], this could then be another mechanism by which cells actively maintain a pool of vicinal extracellular ascorbate to facilitate extracellular ferrireduction, and potentially copper reduction as well.

Thus, in addition to being ascorbate being an important source of reducing equivalents for DCYTB, this protein may also be actively involved in the conservation of the reduced form of the vitamin in the extracellular space. Whether this activity is directly involved in duodenal iron or copper reduction prior to absorption has yet to be examined.

## 3. Coordination of Systemic and Dietary Iron Absorption: Role of the IRP1-HIF2α Axis

### 3.1. The IRP-IRE System

Cellular iron homeostasis is controlled mainly at the post-transcriptional and transcriptional levels. The front-line regulation of cellular iron homeostasis occurs by a post-transcriptional mechanism that controls the rate of synthesis of key iron metabolism proteins involved in iron uptake, storage and release [1,12,108]. The IRP-IRE system is responsible for this post-transcriptional regulation, and allows for rapid alterations in translation of key iron metabolism proteins in response to intracellular iron [1,12,109]. This system depends on the mRNA-binding proteins, IRPs-1 and -2, which post-transcriptionally control the expression of mRNAs possessing IREs [1,12,109]. The IRPs bind to IREs in the 5′- or 3′-untranslated regions (UTRs) of key mRNAs involved in iron metabolism with varying and high affinities in iron-depleted cells [12,14]. When cellular iron levels are increased, which can be potentiated by ascorbate [49], IRP1 molecules increasingly lose their IRE-binding activity by acquiring a (4Fe-4S) ISC [109]. The biogenesis of this ISC within IRP1 occurs within the cytosolic ISC biogenesis (CIA) pathway, and is directly regulated by the activity of the ‘CIA targeting complex’, formed by the CIA ISC targeting proteins, CIA1, CIA2A and MMS19 [110]. The acquisition of this (4Fe-4S) cluster converts IRP1 into a cytosolic aconitase, and thus IRP1’s conversion to the ISC-containing form is dependent on active ISC biogenesis in the mitochondria and/or the cytosol [109]. IRP2 is unable to acquire an ISC [111], and it is instead regulated at the level of protein abundance by proteasomal degradation via the IRP2-targeting E3 ubiquitin ligase subunit, F-box and leucine-rich repeat protein 5 (FBXL5); a protein which itself is regulated at the level of protein stability by iron and oxygen [112,113]. For recent reviews of the IRP/IRE system in iron homeostasis, see [114,115,116].

IRP1 and IRP2 have overlapping functions, as only *Irp1*^−/−^/*Irp2*^−/−^ mice demonstrate embryonic lethality, while singly IRP deficient mice (*i.e.*, *Irp*^−/−^ or *Irp2*^−/−^) remain viable and fertile [117]. Moreover, conditional deletion of both *Irp1* and *Irp2* in hepatocytes and the intestine results in death of affected mice at around 12 days and 4 weeks of age, respectively [118,119]. Furthermore, conditional deletion of both *Irp1* and *Irp2* in murine hepatocytes leads to deficiencies in ISC biogenesis and mitochondrial iron supply, suggesting that the IRPs are crucial for the delivery of iron to mitochondria [119].

*Irp2*^−/−^ mice display dysregulated iron metabolism in the duodenum, CNS and motor neurons of the spinal cord [120]. This dysregulation of iron metabolism is evidenced by an increase in ferritin synthesis, and decrease in TfR1 expression, within the duodenum, CNS and brain neurons [120]. These effects are coupled with pronounced accumulation of iron in the duodenal mucosa and within neurons and oligodendrocytes in distinct brain regions [120]. These findings indicate that IRP2 is critical for regulation of iron metabolism in the duodenum and at least parts of the CNS (for a recent review, see Zhang *et al.* [116]). Crucially, these mice also display a microcytic hypochromic anemia, indicating that IRP2 is also crucial for regulation of iron metabolism in the erythropoietic compartment [121,122]. In these animals, the erythroblasts demonstrate decreased TfR1 expression, leading to decreased TfR1-dependent iron uptake, and increased production of protoporphyrin IX (the iron-devoid heme precursor), which is probably due to increased expression of the erythroid-specific isoform of the first enzyme in the heme synthesis pathway, δ-aminoleuvulinate synthase. The mRNA for this protein possesses an IRE in its 5′-UTR, and its synthesis is consequently upregulated by decreased IRE-binding activity.

While *Irp1*^−/−^ mice at first appeared asymptomatic when maintained on a normal iron diet, it is now clear that IRP1 plays crucial roles in the regulation of systemic iron homeostasis and erythropoiesis (for a recent review, see Zhang *et al.* [116]). Interestingly, *Irp1*^−/−^ mice have a tendency towards higher hematocrits (*i.e.*, polycythemia) in older animals [121,123], as well as pulmonary hypertension, both of which are exacerbated by a low-iron diet [124]. Additionally, serum erythropoietin (EPO) levels of *Irp1*^−/−^ mice are markedly greater (*i.e.*, >7-fold) than in wildtype mice on an iron-deficient diet [124]. Furthermore, these animals exhibit both splenomegaly and increased splenic erythropoiesis, which suggests that loss of IRP1 in these animals leads to the increased renal production of EPO and EPO-dependent extramedullary erythropoiesis (*i.e.*, erythropoiesis occurring outside the bone marrow) [124]. As discussed further below, the molecular basis of this dysregulation of iron metabolism in *Irp1*^−/−^ mice appears to be due largely to an increase in HIF2α expression. Indeed, it now appears that the regulation of HIF2α translation may be the major target of IRP1-dependent regulation of cell iron metabolism, at least in the duodenum and liver, while IRP2 may be responsible for the IRP-dependent regulation of most other IRE-containing transcripts [125].

### 3.2. Emerging Role of the IRP1-HIF2α Axis—Regulation of Erythropoiesis

In addition to post-transcriptional regulation, iron homeostasis is also regulated at the level of the transcription of iron metabolism genes. A major regulator of these changes in transcription is the HIF system, which includes the oxygen- and iron-regulated transcription factors, HIF1α and HIF2α [126]. Low oxygen tensions (*i.e.*, hypoxia), as well as low intracellular iron and ascorbate concentrations, activate HIF1α and HIF2α-regulated transcription by the increased formation and transcriptional activity of heterodimers of HIF1α or HIF2α with the constitutively expressed HIF1β subunit [126]. HIF1α is ubiquitously expressed, while HIF2α has a more restricted tissue distribution [14,127]. The HIFα/β heterodimers form transcription factors that translocate to the nucleus and regulate a range of genes, which possess hypoxia response elements (HREs), encoding proteins important for cellular oxygen homeostasis and the response to hypoxia [126].

Both HIF1α and HIF2α are post-translationally regulated at the level of protein degradation in an oxygen-dependent and iron-dependent manner [126]. This occurs by a specific class of 2-oxoglutarate-dependent dioxygenases: the prolyl-4-hydroxylase domain-containing iron-dependent prolyl hydroxylases (PHDs) 1–3, and the asparaginyl hydroxylase, factor inhibiting HIF1 (FIH1) [127]. However, in the case of HIF2α, the inhibitory effect of FIH1 appears to be minor [128]. Under conditions of iron repletion, these hydroxylases are active under the typical oxygen tensions found under cell culture conditions (*i.e.*, 21%) [127]. These enzymes hydroxylate HIFα proteins at specific proline residues, leading to proteasomal degradation, or at specific asparagine residues, resulting in inactivation of transcriptional activity [127]. Importantly, it is the strict dependence of these hydroxylases on iron that allows cellular iron to modulate HIF-regulated gene expression [127]. The hydroxylated α-subunits of HIF are targeted for ubiquitination by the elongin B-elongin C-cullin-2-von Hippel-Lindau (VHL) E3 ubquitin ligase (also known as CRL-2) subunit, which earmarks the proteins for degradation by the proteasome [126].

HIF2α is the dominant transcription factor *in vivo* that controls renal production of EPO and subsequent erythropoiesis in response to both oxygen concentration and iron levels [129,130,131]. It is now apparent that there is a vital intersection between the IRP/IRE and HIF systems that is pivotal to the regulation of systemic iron homeostasis. Indeed, as alluded to above, recent studies indicate that IRP1, but not IRP2 [123,125,132], is a crucial regulator of HIF2α levels in duodenum, liver and kidney [123,124,125]. This regulation occurs because of an IRE present in the 5′-UTR of the *HIF2α* mRNA, which results in increased translation of HIF2α in response to a decrease in IRP1-IRE binding activity [133]. In *Irp1*^−/−^ mice, the lack of IRP1 in kidney (specifically renal interstitial fibroblasts) leads to a derepression of HIF2α translation, leading to increased HIF2α levels. The increase in HIF2α leads to upregulation of the transcription of the *Epo* gene and increased expression of EPO, which is then secreted and stimulates erythrocyte production [116].

Thus, while oxygen (and iron sensing) by the PHD-regulated proteasomal degradation system regulate HIF2α protein degradation (see above), the IRP1-modulated regulation of HIF2α translation allows for fine-tuning of the level of HIF2α in renal interstitial fibroblasts so that EPO-dependent erythropoiesis does not consume excessive iron [116]. That is, IRP1-dependent sensing of low circulating levels of iron, via decreased production of the ISC in IRP1 and increased IRP1-IRE binding, leads to repression of HIF2α translation and a consequent decrease in *EPO* transcription, ensuring that EPO production does not continue unabated when circulating iron levels become depleted by erythropoiesis.

Importantly, other key HIF2α targets include *DMT1*, *FPN1* and *DCYTB* [134,135,136]. As such, *Irp1*^−/−^ mice show enhanced levels of HIF2α in duodenum, which results in an increase in duodenal DMT1, FPN1 and DCYTB (discussed further in Section 3.3 below). As these animals also display enhanced erythropoiesis due to a HIF2α-dependent increase in renal EPO production [125], these results suggest that the IRP1-HIF2α axis is crucial for coordinating erythropoiesis and dietary iron absorption with iron and oxygen sensing [125].

In summary, the IRP1-HIF2α axis is crucial to the regulation of both iron and oxygen sensing with erythropoiesis and dietary iron absorption [123,125].

### 3.3. Emerging Role of the IRP1-HIF2α axis—Regulation of Duodenal Iron Absorption

Shortly after the discovery of DCYTB, both DCYTB and DMT1 were found to be downregulated in mice shortly after they were provided with a large oral iron bolus [137]. This behavior provides a likely mechanism for the phenomenon of “mucosal block”, which occurs when the absorption of a low dose of iron is downregulated by a preceding large iron bolus provided several hours earlier [137,138,139]. The phenomenon of mucosal block is of great importance to the field of iron homeostasis and iron deficiency, as it pertains to the ability of the duodenum to act as both an important sensor iron levels that can potentially act independently of the hepcidin-FPN1 axis [140,141].

**Figure 2:**
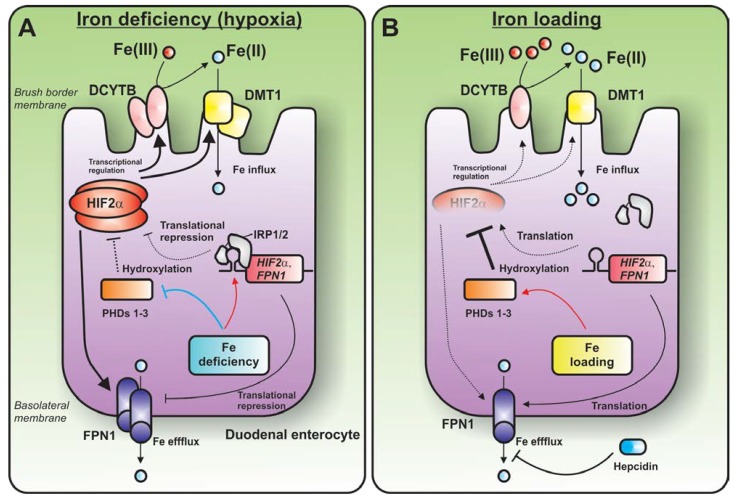
Model for the IRP1-HIF2α axis in regulating DCYTB and dietary iron absorption. (**A**) Under conditions of iron deficiency (as well as hypoxia or increased erythropoietic drive), the expression of HIF2α is increased in the duodenal enterocyte. This leads to transcriptional up-regulation of key iron-metabolism proteins, including DCYTB, DMT1 and FPN1. Specifically, the increase in HIF2α leads to an augmentation of DCYTB and DMT1 in the brush border (*i.e.*, apical) membrane of the duodenal enterocyte and an increase in FPN1 levels in the basolateral membrane. HIF2α has been shown to bind to HRE elements in the regulatory regions of the promoters for DCYTB and DMT1. HIF2α is also regulated at the post-transcriptional level (as a consequence of the IRE in its 5′-UTR) and post-translational level (as a consequence of PHD activity). Under conditions of iron deficiency in the duodenum, although the increased IRE-binding activity of the IRP1 would tend to decrease HIF2α translation, the stabilizing effect of low PHD activity on HIF2α protein levels may predominate, leading to a net increase in HIF2α and a consequent up-regulation of DCYTB and DMT1. (**B**) In contrast, under conditions of high iron in the duodenum, HIF2α is translationally derepressed by IRP1, yet PHD activity is likely to be increased, leading to increased HIF2α hydroxylation and proteasomal degradation. The net effect is a decrease in HIF2α levels, resulting in decreased DCYTB, DMT1 and perhaps FPN1 levels.

How is DCYTB regulated by duodenal iron levels? The initial answer to this question came from two studies published in 2009 that indicated that DCYTB and DMT1 can be up-regulated via HIF2α-dependent transcription of their respective genes [134,135] (Figure 2). Unlike the +IRE isoforms of DMT1 (the main isoform expressed in the duodenum), *DCYTB* mRNA contains no known IREs and cannot be directly regulated by the IRP-IRE system. These studies indicate that HIF2α is a crucial regulator of duodenal iron absorption that may operate independently of the systemic iron homeostatic regulators (e.g., hepcidin) [134,135,140].

FPN1 [136] is also positively regulated by HIF2α-dependent transcription, although it can also be regulated by the IRP-IRE system in virtue of the IRE in its 5′-UTR [134]. An important complicating factor to this story is that, as a consequence of the IRE in its 5′-UTR, HIF2α is itself post-transcriptionally regulated by IRP1 in the duodenal epithelium [123,125]. This is in addition to the iron-dependent regulation of HIF2α at the post-translational level by the PHDs [140]. Thus, the IRP1-HIF2α axis appears to be a crucial regulator of DCYTB in duodenal enterocytes and an important contributor to the mechanism by which dietary iron absorption is regulated by dietary iron and hypoxia (Figure 2). This has important ramifications for our understanding of the mechanisms of iron deficiency anemia and the possible role of DCYTB in this condition.

How does the IRP1-HIF2α axis regulate iron absorption genes *in vivo*? Under conditions of iron deficiency, hypoxia or increased erythropoietic drive, HIF2α levels are increased in the duodenal epithelium [140,142]. This leads to transcriptional up-regulation of key iron-metabolism proteins, including DCYTB, DMT1 and FPN1 [134,135,136,142]. Specifically, the increase in HIF2α leads to an increase in DCYTB and DMT1 in the brush border (*i.e.*, apical) membrane of the duodenal enterocyte [134] and an increase in FPN1 levels in the basolateral membrane [136]. At least in the case of DCYTB and DMT1, HIF2α has been shown to bind to HRE elements in the regulatory regions of the promoters in their respective genes [134]. HIF2α is regulated at the post-transcriptional level as a consequence of the IRE in its 5′-UTR, and at the post-translational level as a consequence of PHD activity [140]. Under conditions of iron deficiency in the duodenum, although the increased IRE-binding activity of the IRPs would tend to decrease HIF2α translation, the stabilizing effect of low PHD activity may predominate, leading to a net increase in HIF2α and a consequent up-regulation of DCYTB and DMT1 [140] (Figure 2A). In contrast, under conditions of high iron in the duodenum, HIF2α is translationally derepressed by the IRP-IRE system, yet PHD activity is likely to be increased [140] (Figure 2B). This response may lead to a net decrease in HIF2α (as a consequence of the enhanced activities of the PHDs) resulting in decreased DCYTB and DMT1 levels. Additionally, under conditions of hypoxia, both HIF2α and DCYTB increase within several hours of hypoxia, while a change in hepcidin expression, which is also regulated by the HIF system, is increased by 72 h [142]. These latter findings suggest that HIF2α predominates over hepcidin in the regulation of intestinal iron absorption during short hypoxic stimulation, and that the intestine exerts regulation over dietary absorption that is independent of the hepcidin-FPN1 axis [142].

## 4. Conclusions

In this review, we have discussed the emerging role of DCYTB and ascorbate in iron absorption and iron homeostasis. Iron and ascorbate are vital cellular constituents in mammalian systems, with the bulk-requirement for iron being during erythropoiesis. Iron homeostasis is controlled at the level of uptake, rather than excretion, and accumulating evidence strongly suggests that in addition to the known ability of dietary ascorbate to enhance non-heme iron absorption in the gut, ascorbate regulates iron homeostasis. While the mechanisms of ascorbate’s involvement in dietary iron absorption are unclear, ascorbate appears to be involved in the provision of electrons to a family of trans-membrane redox enzymes, namely those of the cytochrome *b*_561_ class. These hemoproteins oxidize a pool of ascorbate on one side of the membrane in order to reduce an electron acceptor (e.g., non-heme iron) on the opposite side of the membrane. One crucial member of this family, DCYTB, appears to play an important role in reducing non-heme iron in the gut prior to uptake by ferrous-iron transporters. Accumulating evidence suggests a scenario in which DCYTB is regulated by HIF2α in coordination with IRP1 and PHDs in the duodenal mucosa, particularly in response to iron deficiency and hypoxia. The vital role of DCYTB in dietary iron absorption has important ramifications for our understanding of dietary iron absorption, systemic iron homeostasis and the contributions of DCYTB and ascorbate to iron deficiency anemia.
